# Beyond excision: modern plastic surgery techniques for hidradenitis suppurativa reconstruction

**DOI:** 10.1080/23320885.2025.2583879

**Published:** 2025-11-21

**Authors:** Ruben Sanchez Eligio, Christopher J. Salgado

**Affiliations:** ^a^ Department of Plastic Surgery, Larkin Community Hospital Palm Springs Campus, Hialeah, FL, USA; ^b^ Private Practice; Faculty, Larkin Community Hospital, Constructive Surgery, Miami, FL, USA

**Keywords:** modern plastic surgery techniques, suppurative hidradenitis, regenerative medicine, wound reconstruction, functional restoration

## Abstract

Hidradenitis suppurativa (HS) is a chronic inflammatory skin disorder characterized by recurrent abscesses, sinus tract formation and extensive scarring. In severe cases, surgical excision and complex reconstruction are often required. This case report aims to highlight the role of modern plastic surgical techniques, including regenerative technologies, in managing severe HS. A 37-year-old Hispanic male with a 17-year history of HS presented with extensive lesions involving the buttocks, groin, genitalia, thighs and perianal region. He underwent staged wide excisions totaling over 2,400 cm^2^. Reconstruction included split-thickness skin grafts (STSG), NovoSorb^®^ BTM (Biodegradable Temporizing Matrix) and RECELL^®^ autologous skin cell suspension. Postoperative recovery was marked by successful graft take, wound healing and return to normal function, including physical activity and sexual function, by postoperative day 69. This case underscores both the complexity of managing stage III HS and the evolving role of regenerative technologies in improving outcomes. While wide local excision remains the cornerstone of treatment for extensive disease, adjunctive use of BTM and RECELL enhances dermal regeneration, reduces donor-site morbidity and optimizes aesthetic and functional results. These innovations reflect a shift in reconstructive strategy, emphasizing a more tailored, patient-centered approach. The integration of regenerative modalities such as Biodegradable Temporizing Matrix (BTM) and RECELL autologous cell suspension technology into contemporary plastic surgical reconstruction offers significant benefits in treating severe hidradenitis suppurativa. By complementing traditional excisional techniques, these technologies contribute to improved healing, minimized morbidity and restored function, aligning with the goals of modern, multidisciplinary HS management.

## Introduction

Hidradenitis suppurativa (HS) is a chronic, inflammatory, debilitating dermatologic condition characterized by recurrent painful nodules, abscesses and sinus tract formation. In advanced stages, it often leads to extensive scarring, tissue destruction and functional impairment, significantly impacting quality of life. In such severe cases, radical surgical excision becomes necessary.

In recent years, biologic therapies have become a cornerstone in the management of moderate to severe HS. Adalimumab has shown a clinical success rate of approximately 60%, though many patients experience inadequate or diminishing responses. The recent approval of secukinumab, an anti-IL-17A monoclonal antibody, marks a major advancement in the therapeutic landscape. Other emerging biologics, such as spesolimab and povorcitinib, are currently under investigation, reflecting the growing need for targeted therapies. Among these, secukinumab stands out as the most immediate and promising option for patients with an inadequate response to conventional treatments [1].

Surgical intervention remains essential in managing severe, refractory cases. Plastic and reconstructive surgery plays a vital role in restoring both form and function, with modern techniques aimed at minimizing donor-site morbidity and optimizing aesthetic and functional outcomes. Innovative wound management strategies, such as the use of Biodegradable Temporizing Matrix (BTM) and the RECELL autologous cell suspension system, have expanded reconstructive options. BTM is a synthetic dermal substitute designed to facilitate neodermis formation in complex wounds, while RECELL enables the application of autologous skin cell suspensions to accelerate re-epithelialization and improve healing outcomes.

In this article, we present a case of severe HS treated with extensive local excision followed by reconstructive surgery, highlighting the value of a multidisciplinary approach in achieving favorable results.

### Case presentation

**Figure 1.**
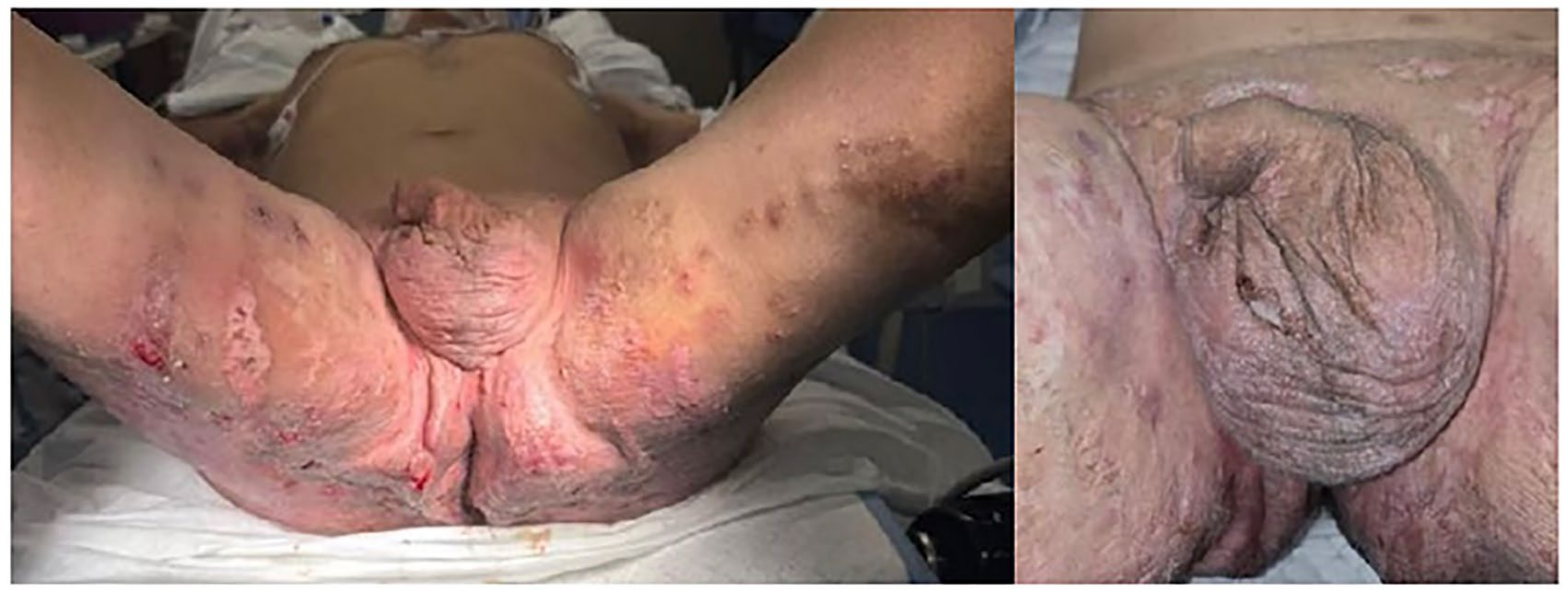
Preoperative pictures with diffuse involvement, widespread scarring and purulent discharge.

A 37-year-old Hispanic male, BMI 24.2, non-smoker, presented with a 17-year history of painful extensive lesions involving the buttocks, groin, genitalia, thighs and perianal region (Figure 1). He was diagnosed with hidradenitis suppurativa and, after clinical evaluation, was admitted on 22 November 2024 for surgical treatment of HS affecting the bilateral thighs, groin and buttocks.

### Surgical procedures

**Figure 2.**
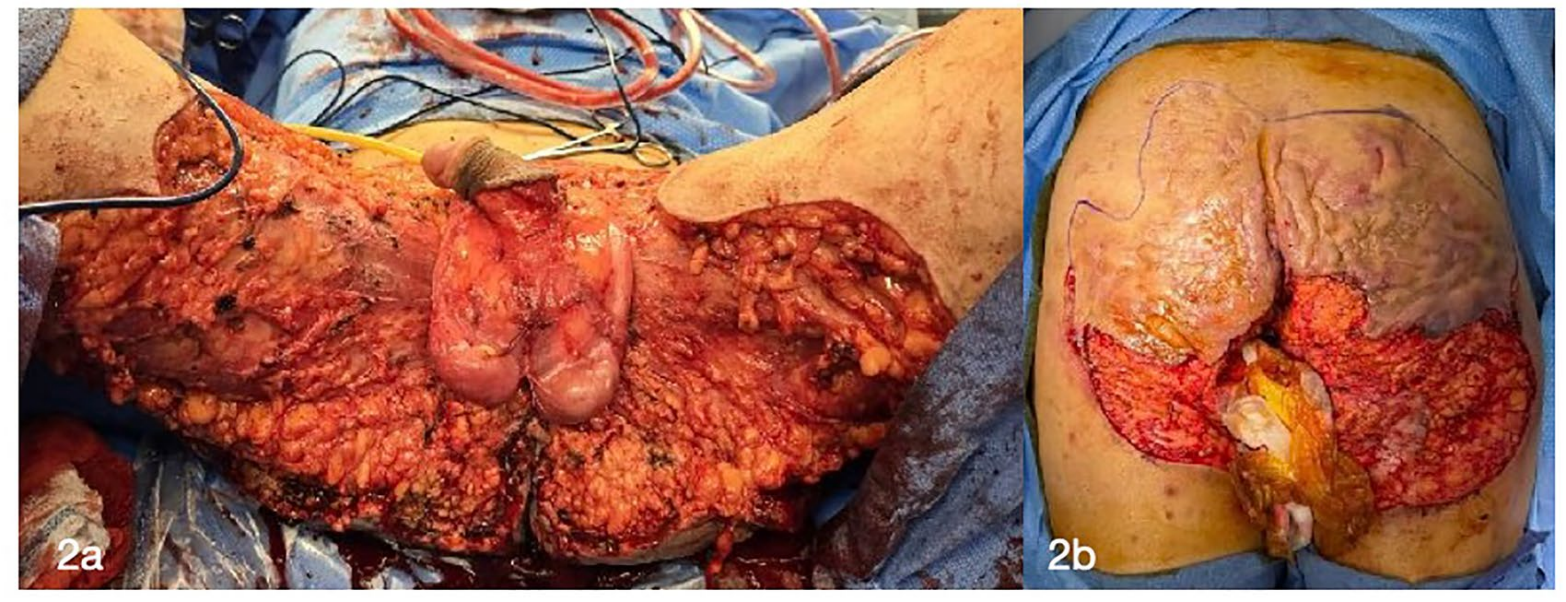
Wide local excision area anterior (a) and posterior (b) markings of the buttock excision site.

**Figure 3.**
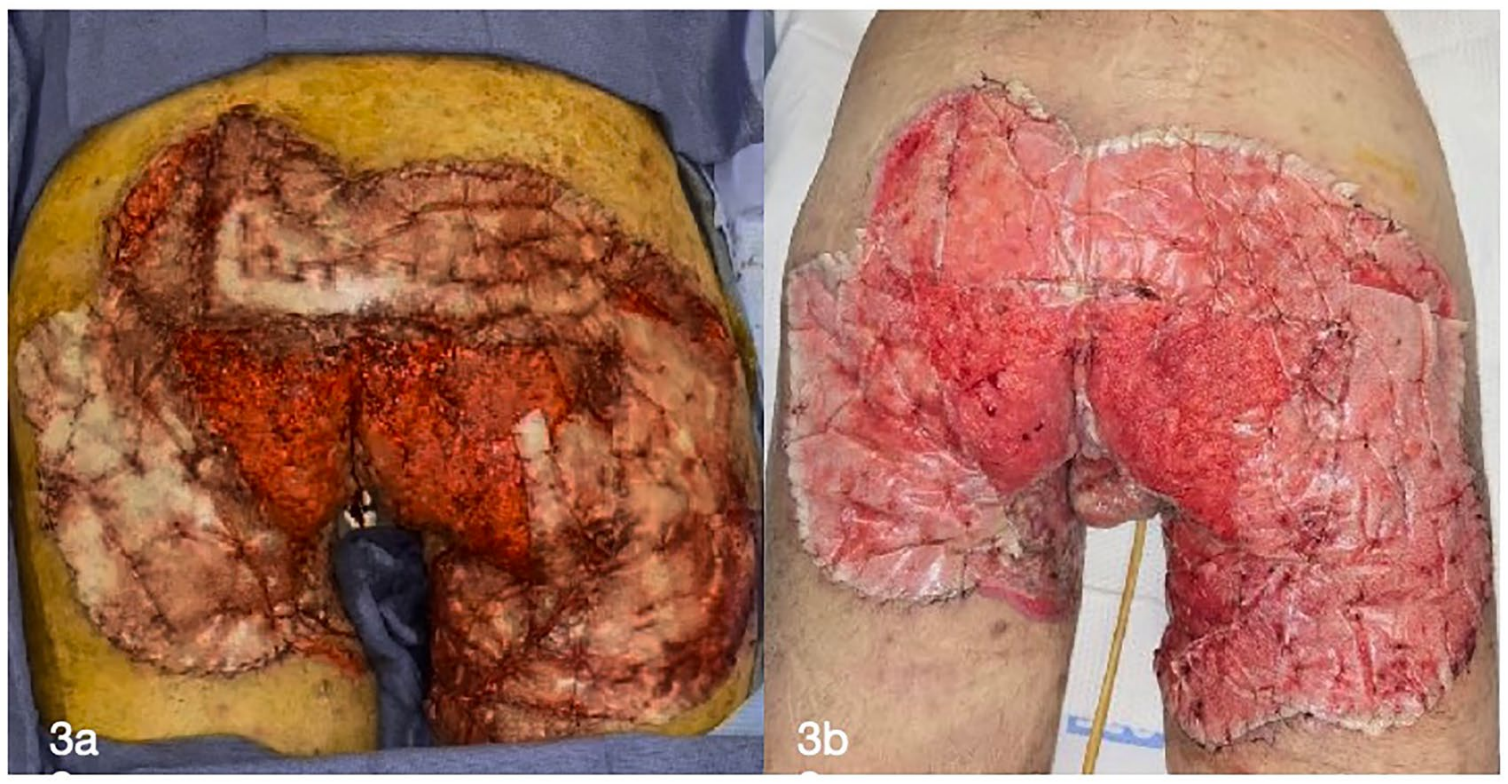
NovoSorb^®^ BTM (Biodegradable Temporizing Matrix) application (a) and postoperative picture (b).

**Figure 4.**
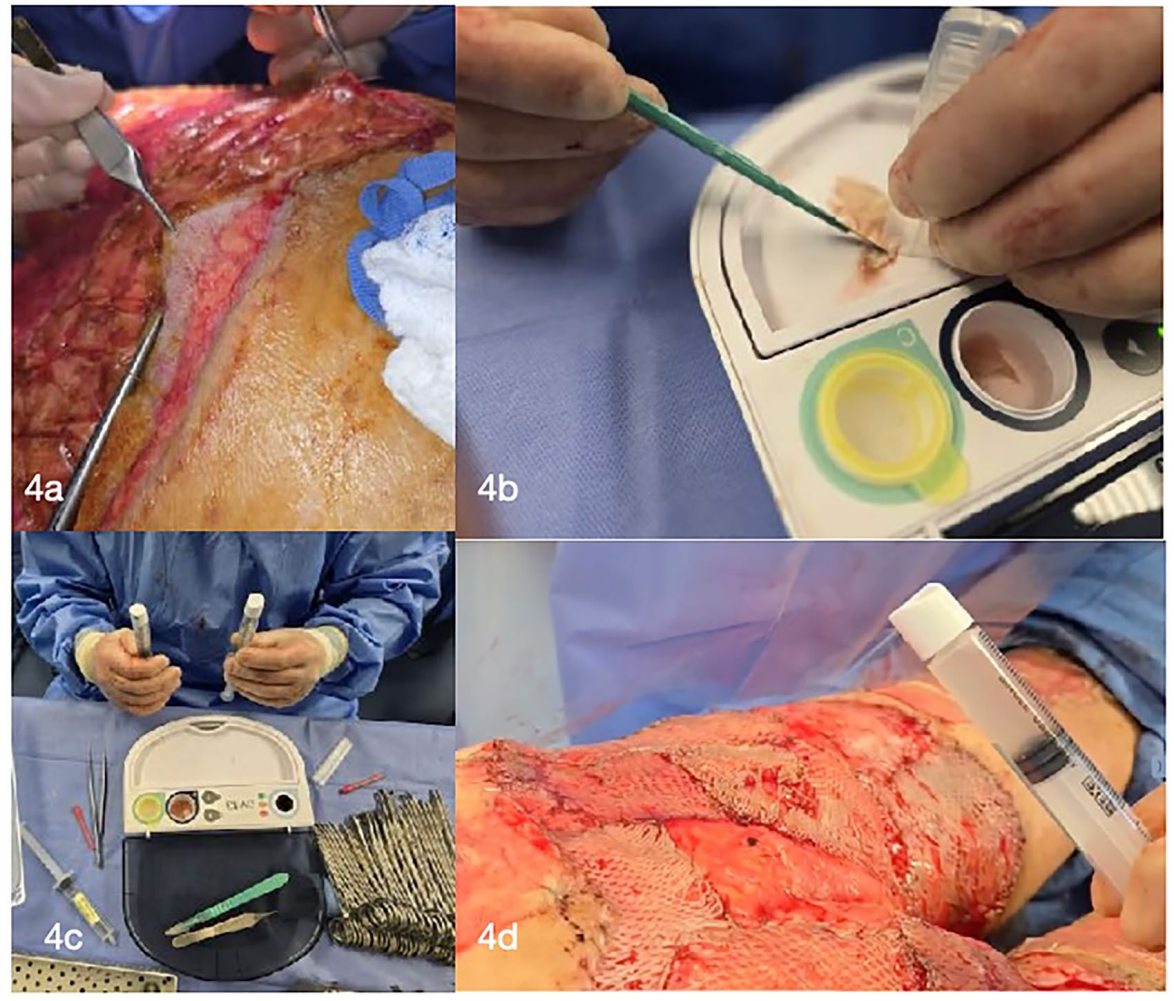
Removal of the NovoSorb^®^ BTM (Biodegradable Temporizing Matrix) sealing membrane, revealing a well-vascularized granulation tissue bed (a). Intraoperative preparation and application of RECELL^®^ autologous skin cell suspension, administered *via* spray technique over meshed split-thickness skin grafts to enhance re-epithelialization and graft integration (b, c and d).

**Figure 5.**
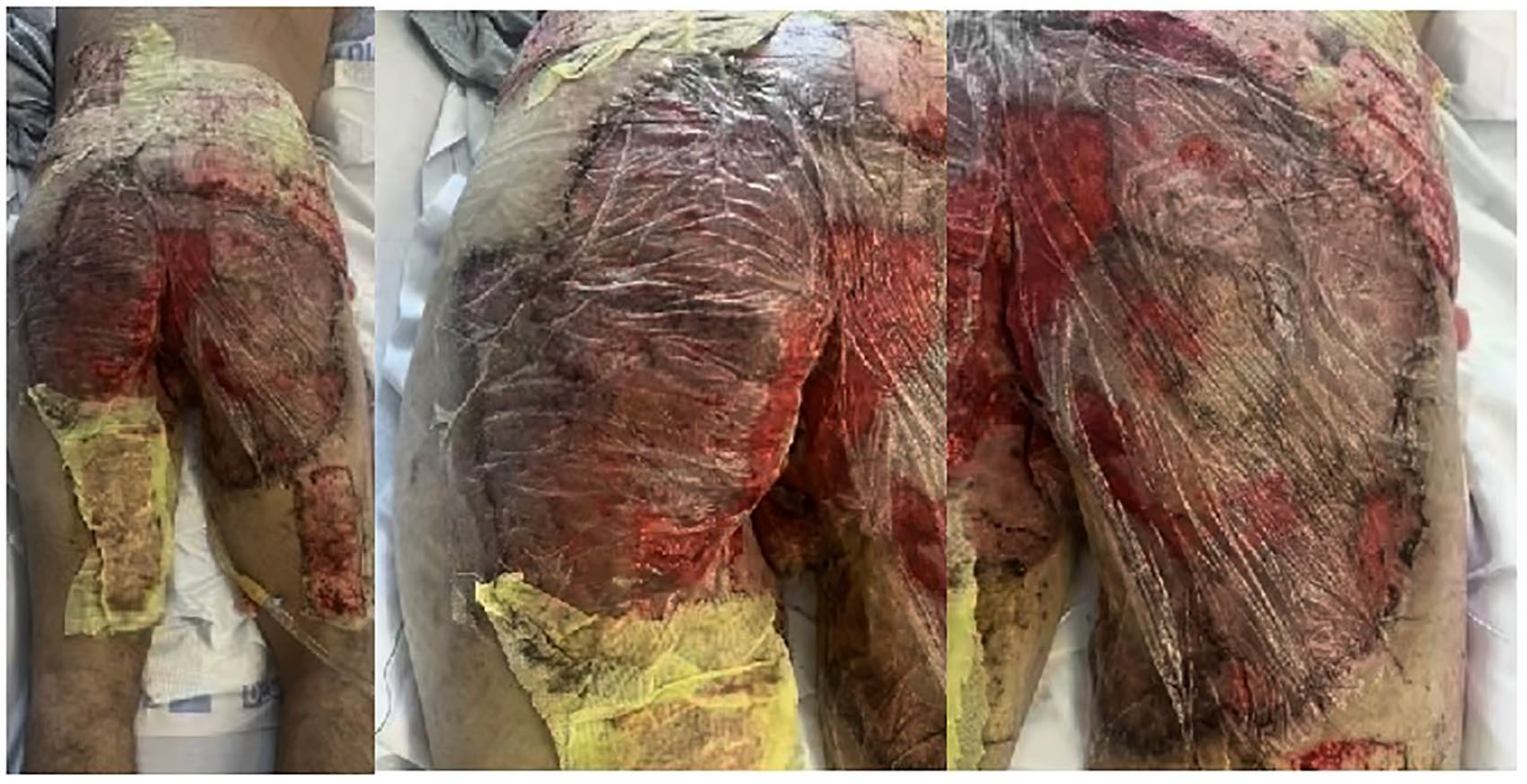
After application of RECELL^®^ cell suspension a non-adherent, non-absorbent, small pore dressing was placed over it. A secondary dressing moderately absorbent, minimally adherent, low shear was then placed over the primary dressing.

**Figure 6.**
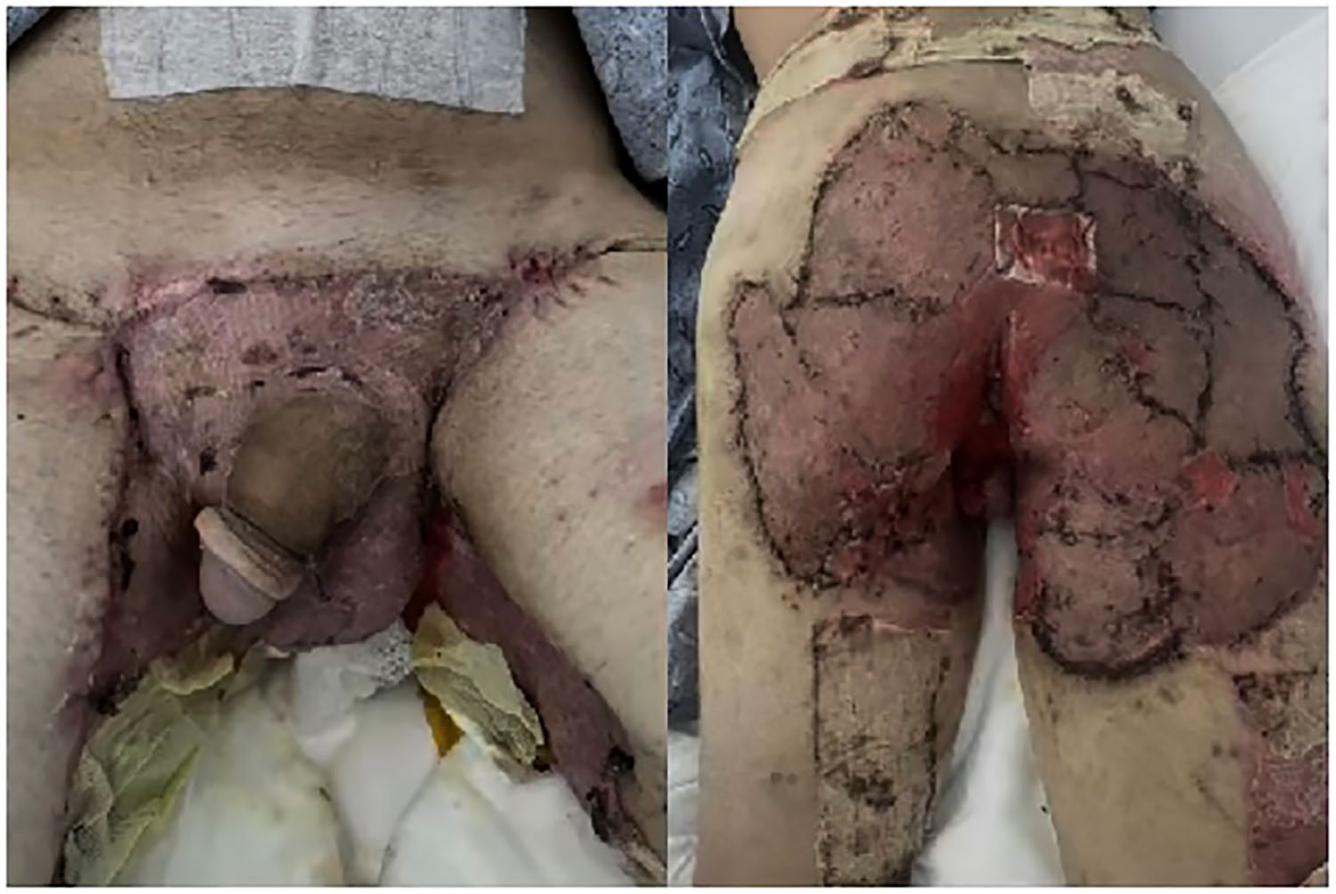
Postoperative image taken during admission showing successful graft integration.

- **22 November 2024:** Under general anesthesia, wide local excision was performed. The resection involved the entire buttock region, starting with a scrotectomy and proximal flap skin resection. Extensive purulent collections were encountered. The total resection measured approximately 1800 cm^2^. Cultures obtained 11/22 grew ESBL-producing E. coli, treated with meropenem.
- **25 November 2024:** Second wide local excision was performed, extending from the buttocks, perianal region with preservation of external anal sphincter, lower back, posterior thighs, scrotum and groins. A small portion of the penile skin was preserved. Extensive purulent drainage was noted. Resection totaled approximately 600 cm^2^ (Figure 2).

- **02 December 2024:** Skin grafting was performed to the abdomen, phallus, testicles and medial thighs and secured using chromic sutures and staples. A total of 450 cm^2^ of skin graft was harvested and fixed to recipient sites, with antimicrobial solution (Irrisept) used for wound irrigation prior to placement. Xeroform and A-Cell were used on the donor sites.
- **09 December 2024:** Under general anesthesia, after negative cultures were obtained in 12/02, new debridement of the bilateral gluteal region and posterior thighs was performed, followed by irrigation with antimicrobial solution (Irrisept). Biodegradable temporizingmatrix (BTM; NovoSorb was then applied to the wounds and sutured in place (Figure 3).

- **12 December 2024:** The patient had successful healing of prior grafts but required additional skin grafting for the buttocks and lower back. The biological tissue matrix external layer was removed, revealing excellent granulation tissue. Skin grafts harvested from the lower back and bilateral thighs were meshed (1:1.5) and supplemented with autologous epidermal culture suspension (RECELL). Grafts were sutured with 4-0 chromic suture and covered with RECELL adhesive barrier. Xeroform was applied to the donor sites (Figures 4 and 5).

- **19 December 2024:** Under general anesthesia, staples were removed from the groins. The graft was found to be taking properly. The patient was then repositioned prone, and the RECELL adhesive barrier was removed, revealing an intact graft. A small remaining area in the sacral region was treated with BTM and fixed with chromic suture (Figure 6).

- **23 December 2024:** The patient’s recovery was uneventful, with satisfactory healing of wounds. The patient was discharged home with post-operative care instructions: out of bed with assistance, avoiding direct sitting, daily dressing changes of dorsal wounds; grafted areas to remain open to air, protein supplementation to optimize wound healing and follow-up in clinic one week after discharge.

### Postoperative follow-up

**Figure 7.**
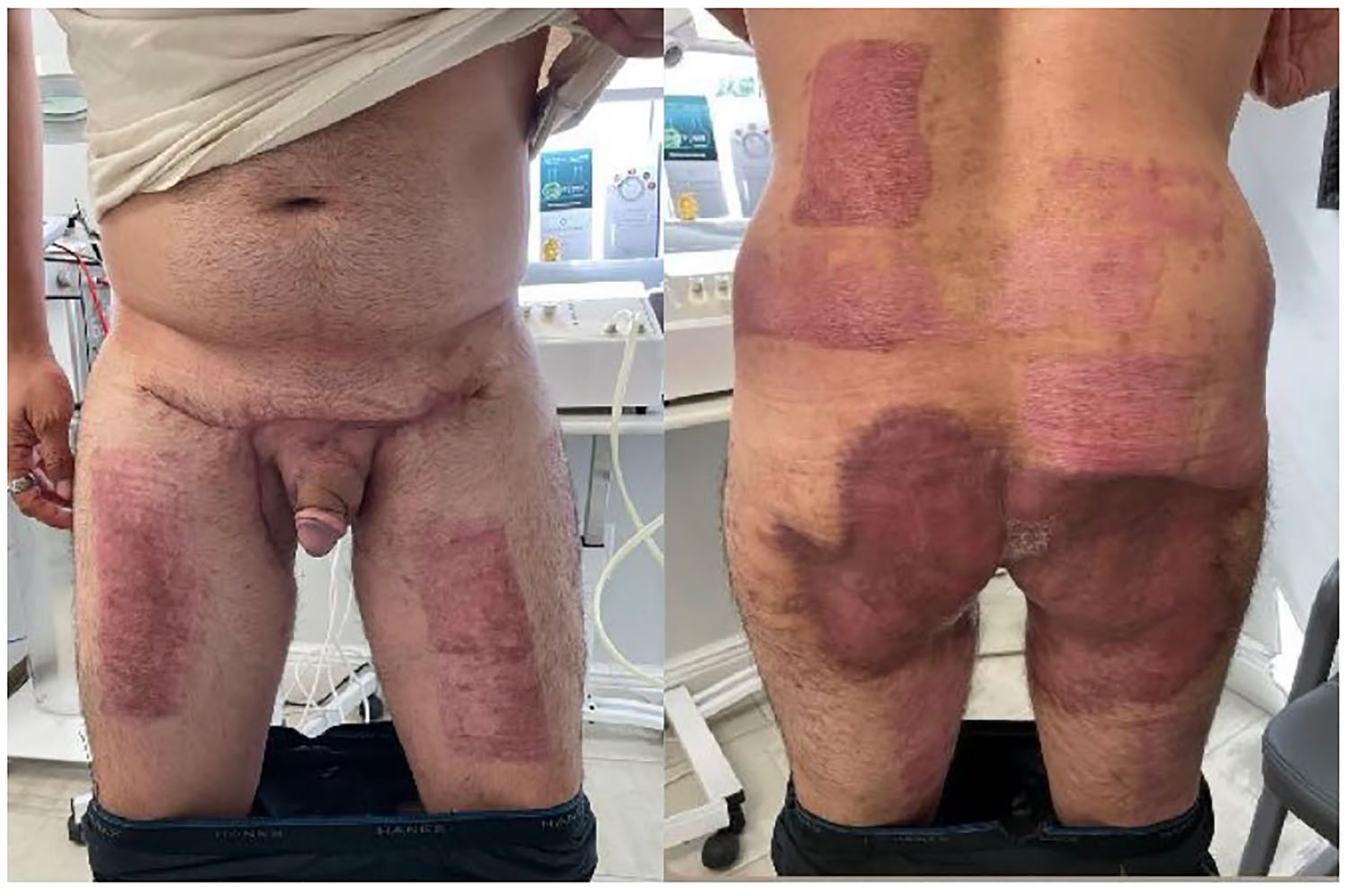
Postoperative day 69 demonstrating surgical and donor sites with satisfactory healing.

- **Postoperative day (POD) 27 Visit 1 (08 January 2025):** Patient presented with well-healed STSG in the groin area with hypergranulation tissue noted on the posterior buttocks and left groin with no signs of infection. Silver nitrate was applied to hypergranulation tissue, local wound care with Bacitracin and abdominal pads was recommended.
- **POD 41 Visit 2 (22 January 2025):** Hypergranulation tissue showed a gradual reduction. A new application of silver nitrate was administered, followed by the application of Aquaphor to both the donor and split-thickness skin graft (STSG) sites.
- **POD 69 Visit 3 (19 February 2025):** The patient was actively participating in daily activities and workouts without any complaints. He reports normal erections with slight tightness but no pain. Mild penile swelling, attributed to impaired lymphatic drainage, was observed. There are no open wounds, scar contractures, or limitations in mobility. The patient has been cleared for water submersion and all activities, including sexual activity. Scar massage at the base of the penis was recommended to help reduce swelling and soften the area. Postoperative recovery has been steady, with proper wound healing, resolution of hypergranulation tissue and no signs of HS recurrence. Minor issues such as edema and tightness are being managed with conservative treatment (Figure 7).

## Discussion

Hidradenitis suppurativa (HS), also referred to as acne inversa, is a chronic, relapsing inflammatory skin disorder characterized by recurrent painful nodules, abscesses, sinus tract formation and scarring. Manifestation occurs between puberty and age 40, with a female-to-male ratio of approximately 3:1. Prevalence ranges between 1% and 4% of the population [2].

The pathogenic event in HS is follicular occlusion, initiating a cascade of inflammation leading to abscess formation and destruction of the pilosebaceous unit and surrounding tissues. Secondary bacterial infections can exacerbate disease progression [2].

Clinically, HS presents with deep painful nodules that may rupture, form sinus tracts and produce chronic draining lesions. With disease progression, it often results in thickened, fibrotic skin, leading to contractures and deformities. The axillae are the most frequently involved sites, but the groin, umbilical region, perineum and inframammary areas are also commonly affected. In advanced cases there is significant fibrosis, disfigurement and functional impairment, often necessitating wide surgical excision. Plastic and reconstructive surgery is pivotal in restoring form, function and quality of life for these patients [3].

Management strategies depend on disease severity, often guided by the Hurley staging system. It is composed of three stages. Stage 1 with single or multiple abscesses without sinus tracts or scarring. Stage 2 with recurrent lesions, single or multiple widely separated with limited sinus formation and scarring and stage 3 with diffuse involvement with interconnected sinus tracts and widespread scarring across an entire area. Regardless of the disease stage, lifestyle modification is a cornerstone of hidradenitis suppurativa (HS) management. Essential measures include smoking cessation, weight reduction, minimizing skin trauma and avoiding tight-fitting or synthetic clothing [3].

Managing HS is particularly challenging. A multimodal treatment strategy is usually required, combining medical therapy with surgical intervention. While medical management—including antibiotics, biologics, hormonal therapy and corticosteroids—can control inflammation and prevent new lesion formation, surgery remains the only option capable of achieving long-term remission. However, recurrence rates vary depending on the surgical technique and the use of adjunct medical therapies. Minimally invasive options such as laser therapy (e.g. Nd:YAG for hair removal and lesion reduction) and surgical interventions are used based on disease severity and lesion type. Incision and drainage are typically reserved for acutely painful, fluctuant abscesses. Similarly, deroofing and local excision carry high recurrence rates. In contrast, wide surgical excision (most often indicated in Hurley Stage III disease) offers a more definitive solution but frequently results in significant tissue defects that necessitate complex reconstructive procedures [3].

Reconstruction options following wide excision include primary closure, split-thickness skin grafting and local or regional flaps. In the presence of active infection, a staged approach using negative-pressure wound therapy (NPWT) to optimize the wound bed prior to delayed reconstruction is commonly performed.

Severe HS presents unique surgical challenges, requiring individualized reconstructive strategies. Multidisciplinary collaboration is critical for optimal outcomes, with attention to preoperative planning and postoperative wound care.

Recent advances in reconstructive techniques have expanded the surgical armamentarium for HS. Examples include extracellular matrix grafts such as ovine forestomach matrix (OFM), used in products such as Myriad Matrix^™^, which has shown promise in supporting soft tissue regeneration following wide excision. Clinical results suggest favorable integration and healing in complex HS cases [4]. Another option is the Skin-Tissue-Sparing Excision with Electrosurgical Peeling (STEEP) technique, a tissue-preserving technique designed to remove diseased areas while maintaining as much healthy tissue as possible, that may improve recovery times and cosmetic outcomes [5]. Another example is CO_2_ laser therapy that ablates affected tissue with precision, promoting healing and reducing recurrence. It may be used alone or in combination with other surgical methods [6].

These innovations highlight the evolving landscape of HS treatment, offering patients a broader range of reconstructive options that can be tailored to disease severity and individual needs.

For severe cases like the one we present, a combination of wide excision, skin grafting and advanced biomaterials (NovoSorb BTM, RECELL autograft) offers a promising reconstructive approach. Advanced reconstructive options using NovoSorb BTM (Biodegradable Temporizing Matrix) for dermal regeneration before grafting and RECELL Autograft (spray-on skin) to enhance wound healing present modern options during reconstruction.

The RECELL autologous cell harvesting device is an innovative medical technology designed to facilitate rapid healing of skin injuries by utilizing a patient’s own skin cells creating a regenerative autologous skin cell suspension (ASCS) from a small sample of the patient’s skin and applying to the wound site to promote healing [7]. The device is indicated for full-thickness skin defects resulting from traumatic avulsion injuries (e.g. degloving), surgical excisions (e.g. necrotizing soft tissue infections), or resections (e.g. skin cancer) in patients aged 15 and older [8].

One of the significant benefits of RECELL is its ability to minimize the need for extensive donor skin harvesting. The device can achieve up to a 97.5% reduction in donor skin requirements compared to traditional autografting methods, utilizing a 1:80 expansion ratio. This means that a 1 cm^2^ donor site can treat an area up to 80 times its size [9]. Clinical studies have demonstrated that RECELL is a safe and effective treatment achieving short- and long-term healing outcomes comparable to standard skin grafting techniques while significantly decreasing donor skin usage [10]. Its application in managing hidradenitis suppurativa (HS) is emerging but not yet widely established.

A case study published with combined use of Integra (biological dermal matrix) and RECELL in severe HS demonstrated successful wound healing without complications, suggesting that integrating RECELL with other advanced wound care products may be beneficial in complex HS cases [11]. In summary, RECELL presents a potential adjunctive treatment for HS, especially in severe cases requiring extensive surgical intervention.

NovoSorb^®^ Biodegradable Temporizing Matrix (BTM) is a synthetic, biocompatible dermal scaffold developed by PolyNovo for the treatment of surgical wounds, partial and full-thickness wounds where the dermal structure has been lost due to trauma or surgical debridement [12,13].

It has a bilayer design with a sealing membrane (temporary, non-biodegradable layer that closes the wound, limiting moisture loss and serving as a barrier to external bacteria) and a matrix (2 mm bioabsorbable open-cell polyurethane foam that allows for cellular infiltration, aiding in the reconstruction of the dermis) [12].

This matrix has associated key features such as infection resistance (unlike biologic alternatives used for HS reconstruction in the past, NovoSorb BTM’s synthetic composition is not a nutrient source for bacteria, making it robust in the presence of infection), minimized scarring and contracture (facilitating the regeneration of a neodermis), cost-effective (typically less expensive than biologic alternatives), ability to be stored at room temperature (≤ 25 °C), available in large sizes (up to 20 × 40 cm), and can be easily applied using sutures or staples [12].

NovoSorb BTM has been utilized in managing challenging wounds, including major burns, necrotizing soft tissue infections, chronic wounds and non-graftable wound beds. Its ability to provide a framework for organized neodermis growth and resistance to infection has been highlighted in clinical settings [14].

In the context of hidradenitis suppurativa (HS), a chronic inflammatory skin condition characterized by recurrent abscesses and sinus tracts, NovoSorb BTM has emerged as a valuable reconstructive option, particularly for extensive and challenging wounds resulting from surgical excision of affected tissues. Traditional reconstruction methods, such as immediate skin grafting or the use of local flaps, may be associated with higher risks of graft loss due to persistent infection, contracture formation and limited range of motion. BTM offers an alternative by providing a temporary dermal scaffold that supports vascularization and integration, potentially leading to improved outcomes [15].

Clinical experiences have demonstrated the efficacy of BTM in HS reconstruction. For instance, a case involving a 49-year-old male with severe bilateral axillary HS who underwent wide excision of the affected area, resulting in a substantial soft-tissue defect. BTM was applied to the wound, followed by negative pressure wound therapy. After approximately four weeks, the sealing membrane was removed, and a split-thickness skin graft was applied. At a one-year follow-up, the patient exhibited successful soft-tissue reconstruction with soft, supple skin and full shoulder range of motion [15].

Another study reviewed the application of BTM across various complex wounds, including those resulting from HS. The findings indicated that BTM could be effectively applied to a wide range of complex wounds, suggesting its potential as an important adjunct in the reconstructive ladder for HS patients [16].

In summary, NovoSorb BTM offers a synthetic alternative to biologic dermal substitutes addressing some limitations associated with biologic materials. Its ability to serve as a temporary dermal scaffold supports vascularization and integration, potentially leading to improved functional and esthetic outcomes. It represents a promising reconstructive option for patients with hidradenitis suppurativa, particularly in cases involving extensive tissue defects. In the case presented, the combined application of RECELL and NovoSorb BTM facilitated accelerated wound healing and effective soft tissue regeneration, supporting their utility in the reconstructive management of extensive hidradenitis suppurativa defects.

## Conclusion

Hidradenitis suppurativa (HS) represents a challenging pathology that, in its severe stages, often necessitates extensive surgical intervention to restore function and quality of life. Traditional reconstructive techniques, while effective, may be limited by high recurrence rates, poor wound healing and donor site morbidity. The proposed combination of NovoSorb Biodegradable Temporizing Matrix (BTM) and the RECELL autologous cell harvesting device used during this case is a valuable option to expand the surgical armamentarium by providing innovative solutions for dermal regeneration and epithelial coverage. Together, these modalities present a synergistic approach to managing complex HS wounds, offering improved healing outcomes, minimized scarring, enhanced functional recovery and cost-effectiveness. The integration of these advanced technologies into a multidisciplinary treatment paradigm marks a significant step forward in the comprehensive care of patients with severe HS.
